# Risk Factors for Multiple Organ Dysfunction Syndrome in Severe Stroke Patients

**DOI:** 10.1371/journal.pone.0167189

**Published:** 2016-11-28

**Authors:** Wei Qin, Xiaoyu Zhang, Shuna Yang, Yue Li, Junliang Yuan, Lei Yang, Shujuan Li, Wenli Hu

**Affiliations:** Department of Neurology, Beijing Chaoyang Hospital, Capital Medical University, Beijing, China; University of Toronto, CANADA

## Abstract

**Background:**

Severe stroke patients have poor clinical outcome which may be associated with development of multiple organ dysfunction syndrome (MODS). Therefore, the aim of our study was to investigate independent risk factors for development of MODS in severe stroke patients.

**Methods:**

Ninety seven severe stroke patients were prospective recruited from Jan 2011 to Jun 2015. The development of MODS was identified by Sequential Organ Failure Assessment (SOFA) score (score ≥ 3, at least two organs), which was assessed on day 1, 4, 7, 10 and 14 after admission. Baseline characteristics, Acute Physiology and Chronic Health Evaluation (APACHE) II score, Glasgow coma score (GCS) and cerebral imaging parameters were collected at admission. Cox regression was performed to determine predictors for the development of MODS. Medical complications after admission and in-hospital mortality were also investigated.

**Results:**

33 (34%) patients were in MODS group and 64 (66%) were in non-MODS group within 14 days after admission. Patients in MODS group had more smoker (51.5% vs 28.1%, *p* = 0.023), higher NIHSS score (23.48 ± 6.12 vs 19.81 ± 4.83, *p* = 0.004), higher APACHE II score (18.70 ± 5.18 vs 15.64 ± 4.36, *p* = 0.003) and lower GCS score (6.33 ± 2.48 vs 8.14 ± 2.73, *p* = 0.002). They also had higher rate of infarction in multi vascular territories (36.4% vs 10.9%, *p* = 0.003). The most common complication in all patients was pulmonary infection, while complication scores were comparable between two groups. Patients with MODS had higher in-hospital mortality (69.7% vs 9.4%, *p* = 0.000). In Cox regression, NIHSS score (RR = 1.084, 95% CI 1.019–1.153) and infarction in multi vascular territories (RR = 2.345 95% CI 1.105–4.978) were independent risk factors for development of MODS.

**Conclusions:**

In acute phase of stroke, NIHSS score and infarction in multi vascular territories predicted MODS in severe stroke patients. Moreover, patients with MODS had higher in-hospital mortality, suggesting that early identification of MODS is critical important.

## Introduction

Although advances in prevention and treatment over the last two decades, stroke remains the major cause of disability and mortality worldwide. Severe strokes account for 2%-12.7% of all ischemic strokes, and are associated with poor short-term and long-term outcome.[1, 2] They are usually termed as malignant middle cerebral artery infarctions (MI-MCA) if total MCA territory is infarcted.[3] The mortality of severe stroke patients is about 36.5% at discharge and 45% at one year follow-up.[2] Only 28% of severe stroke patients present with favorable outcome at 3 months after stroke (modified Rankin scale score of ≤ 3).[2] In addition, patients with severe stroke have a higher risk of neurological deterioration and requiring management in intensive care unit (ICU) because of focal edema with herniation and systemic complications, which could increase hospitalization costs.[4]

It has been known that multiple organ dysfunction syndrome (MODS) is the leading cause of mortality in critically ill patients, which is defined as progressive dysfunction of two or more organ systems following an acute threat to systemic homeostasis.[5] Within the first few weeks of severe stroke, patients may develop MODS because of inappropriate generalized inflammatory response and medical complications, such as brain edema, chest infection, congestive heart failure, and gastrointestinal bleeding.[6] MODS has been an important cause of death in severe stroke patients in ICU. It is estimated that 14% of all patients admitted to ICU develop MODS during their stay, and the syndrome is responsible for about 80% of all deaths in the ICU.[7] Therefore, early identification of patients with MODS is critical important and immediate treatment may help to improve their outcome. However, studies about MODS in severe stroke patients are rare. A previous study showed that diabetes mellitus may correlate with the development of MODS in stroke patients.[8] However, number of severe stroke patients in this study was unrevealed and risk factors for MODS were not analyzed. Another study reported that 11.7% of acute stroke patients have risk of MODS, and age, diseased region close to the mean line, Glasgow coma score (GCS), level of blood sugar, blood white cell count and the chronic disease history are associated with MODS.[9] However, their criteria of MODS were not fully illustrated.

Therefore, the aim our study was to determine independent risk factors for the development of MODS in severe stroke patients. We also investigated characteristics and short-term outcome of severe stroke patients with MODS.

## Materials and Methods

### Subject Population

We conducted this study in Beijing chaoyang hospital affiliated to Capital Medical University from Jan 2011 to Jun 2015. Acute ischemic stroke patients admitted to Beijing chaoyang hospital were prospective identified and included from Jan 2011 to Jun 2015. As there is no consensus on how to define severe stroke, severe stroke patients were recruited if all of the following inclusion criteria were fulfilled: (1) cerebral infarction was confirmed by CT or MRI; (2) a National Institutes of Health Stroke Scale (NIHSS) score ≥ 15 at admission; (3) including a score ≥ 1 for NIHSS 1a (level of consciousness); (4) admitted to hospital within 48 hours after symptom onset. Deaths of patients with “do-not-resuscitate” orders were also not considered in our study. Treatments among patients were similar according to guidelines for the early management of adults with ischemic stroke. Institutional review board of Beijing chaoyang hospital affiliated to Capital Medical University approved the study and families of patients provided their written informed consent to participate in this study.

### MODS Diagnosis

We identified the presence and date of MODS by Sequential Organ Failure Assessment (SOFA) score within 14 days after admission. The SOFA score assesses the function of six different organ systems including respiratory (partial arterial oxygen pressure (PaO2)/fraction of inspired oxygen (FiO2)), cardiovascular (blood pressure, vasoactive drugs), renal (creatinine and diuresis), hepatic (bilirubin), neurological (GCS) and haematological (platelet count). The SOFA score was assessed on days 1, 4, 7, 10 and 14 according to the original definitions.[10] The SOFA score was also assessed on the day if patients had clinical deterioration. We defined SOFA score ≥ 3 as severe organ failure in any organ system. MODS were defined as the occurrence of severe organ failure in two or more organ systems on the same day, like in a prior study.[11] Patients were defined as MODS group and non-MODS group by their SOFA score within 14 days after admission.

### Demographic and Clinical Assessment

All included patients underwent a standard data collection protocol and authors had no access to information that could identify individual participants during or after data collection. The clinical information collected included age, sex and risk factors for stroke (history of hypertension, diabetes mellitus, coronary artery disease, stroke or transient ischemic attack, chronic obstructive pulmonary disease, atrial fibrillation, smoking and alcohol use). We also collected body temperature, heart rate, blood pressure, white blood cell and hemoglobin at admission. Severity of stroke at admission was assessed by NIHSS score. In addition, the Acute Physiology and Chronic Health Evaluation (APACHE) II score was calculated to determine systemic severity of illness and GCS was used to assess the conscious state at admission. As magnetic resonance angiography (MRA) and cardiac ultrasonography were not performed in many patients, it was difficult for us to determine the etiology of stroke by the Trial of ORG 10172 in Acute Stroke Treatment (TOAST) criteria.[12] According to the Oxfordshire Community Stroke Project (OCSP) classification, patients were defined as total anterior circulation infarcts (TACI), partial anterior circulation infarcts (PACI), posterior circulation infarcts (POCI), or lacunar infarcts (LACI) based on their clinical syndromes.[13]

### Cerebral Imaging Parameters

Based on CT or MRI, territories of infarction were identified by two neurologist blinded to their SOFA score and clinical information, and discrepancy in readings was resolved by consensus. They were consisted of territory of anterior cerebral artery, territory of middle cerebral artery, territory of posterior cerebral artery and territory of basal and vertebral arteries. Patients were defined as infarction in a single vascular territory or infarction in multi vascular territories (≥ 2). We did not include other CT parameters (such as horizontal displacement of the septum pellucidum, pineal shift and ≥ 50% MCA hypodensity) in our study because they are closely associated with time point of CT examination.[14]

### Medical Complications and Clinical Outcome

Medical complications after admission (≤14 days) were determined by an experienced neurologist blinded to their SOFA score and clinical information. According to definitions used in previous studies, seven complications were recorded, including pulmonary infection, urinary tract infection, deep vein thrombosis, stress ulcer, epilepsy, hemorrhagic transformation and myocardial infarction.[15, 16] Score of complications were calculated (each complication for 1 point and 7 for the maximum score). We also recorded the incidence of mechanical ventilation (noninvasive and invasive) and osmotherapy in acute phase of stroke (≤14 days). In-hospital mortality was also observed in all patients.

### Statistic Analysis

All statistics were presented as mean and SD for continuous variables with normal distribution, median and interquartile range for continuous variables with non-normal distribution, frequency and percentages for categorical variables. Univariate analysis was performed between severe stroke patients with MODS and those without. Continuous variables with normal distribution were compared with Student t test with significance set at *p* < 0.05, while Wilcoxon rank sum test for continuous variables with non-normal distribution. Categorical variables were compared by means of x^2^ test or fisher’s exact test. Cox proportional hazards regression was performed to determine predictors for MODS. Analysis was performed with Statistical Package for Social Sciences (SPSS version 16).

## Results

During study period, 1347 stroke patients admitted to hospital and 112 severe stroke patients were identified. 11 patients admitted to hospital over 48 hours after symptom onset and 4 patients with “do-not-resuscitate” order were excluded. Finally, 97 patients were included in this study. Only 3 patients underwent intravenous thrombolysis and 2 patients undertook decompressive surgery. 29 patients died at discharge, in which 22 patients died within 14 days after admission. The mean age was 73.90 ± 12.45 years and 51 (52.6%) patients were male. There were 48 (49.5%) strokes in territory of middle cerebral artery, 26 (26.8%) in territory of basal and vertebral arteries, 2 (2.1%) in territory of anterior cerebral artery, 2 (2.1%) in territory of posterior cerebral artery and 19 (19.6%) strokes in multi vascular territories. The mean NIHSS score at admission was 21.06 ± 5.55 and the mean GCS score was 7.53 ± 2.77. Basic characteristics of the study sample are given in Table 1.

**Table 1:** Basic characteristics between patients in MODS group and non-MODS group.

	All patients	MODS group	Non-MODS group	*p* value
	n = 97	n = 33	n = 64	
Age, years	73.90 ± 12.45	73.64 ± 13.57	74.03 ± 11.95	0.883
Sex, male	51 (52.6%)	18 (54.5%)	33 (51.6%)	0.780
Risk factors for stroke				
Hypertension	75 (77.3%)	26 (78.8%)	49 (76.6%)	0.804
Diabetes	32 (33.0%)	10 (30.3%)	22 (34.4%)	0.686
Stroke/TIA(previous)	35 (36.1%)	12 (36.4%)	23 (35.9%)	0.967
COPD	6 (6.2%)	2 (6.1%)	4 (6.2%)	1.000
Coronary artery disease	33 (34.0%)	9 (27.3%)	24 (37.5%)	0.314
Atrial fibrillation	14 (14.4%)	5 (15.2%)	9 (14.1%)	1.000
Smoking	35 (36.1%)	17 (51.5%)	18 (28.1%)	0.023
Alcohol use	23 (23.7%)	9 (27.3%)	14 (21.9%)	0.554
Body temperature at admission				
Normothermia(<37.6°C)	73 (75.3%)	20 (60.6%)	53 (82.8%)	0.052
Mild hyperthermia (37.6°C—38°C)	10 (10.3%)	5 (15.2%)	5 (7.8%)	
Severe hyperthermia (≥38°C)	14 (14.4%)	8 (24.2%)	6 (9.4%)	
Heart rate at admission, /min	89 ± 26	93 ± 33	87 ± 21	0.378
Systolic BP at admission, mmHg	159 ± 30	158 ± 36	160 ± 26	0.817
Diastolic BP at admission, ^a^ mmHg	81 (73, 93)	80 (67, 94)	82 (75, 92)	0.596
White blood cell, 10^9^/L	11.32 ± 3.45	11.56 ± 3.49	11.20 ± 3.44	0.623
Hemoglobin, g/L	134.25 ± 20.51	133.88 ± 24.39	134.44 ± 18.41	0.900
NIHSS score (at admission)	21.06 ± 5.55	23.48 ± 6.12	19.81 ± 4.83	0.004
APACHE II score (at admission)	16.68 ± 4.85	18.70 ± 5.18	15.64 ± 4.36	0.003
GCS score (at admission)	7.53 ± 2.77	6.33 ± 2.48	8.14 ± 2.73	0.002
Time to image (CT/MRI), ^a^ hours	7(4, 12.5)	7 (4, 10)	7 (4, 11.5)	0.682
OCSP				
TACI	47 (48.5%)	16 (48.5%)	31 (48.4%)	0.023
PACI	19 (19.6%)	2 (6.1%)	17 (28.6%)	
POCI	31 (31.0%)	15 (45.5%)	16 (25.0%)	
Radiologic parameters (CT/MRI)				
Single vascular territory	78 (80.4%)	21 (63.6%)	57 (89.1%)	0.003
multi vascular territories (≥2)	19 (19.6%)	12 (36.4%)	7 (10.9%)	
Score of complications ^a^	2 (1, 2)	2 (1, 3)	2 (1, 2)	0.482
Mechanical ventilation	25 (25.8%)	17 (51.5%)	8 (12.5%)	0.000
Osmotherapy	73 (75.3%)	29 (87.9%)	44 (68.8%)	0.039
Death (In-hospital)	29 (19.9%)	23 (69.7%)	6 (9.4%)	0.000

Abbreviations: MODS = multiple organ dysfunction syndrome; COPD = chronic obstructive pulmonary disease; NIHSS = NIH Stroke Scale; APACHE = Acute Physiology and Chronic Health Evaluation; GCS = Glasgow Coma Scale; OCSP = Oxfordshire Community Stroke Project; TACI = total anterior circulation infarcts; PACI = partial anterior circulation infarcts; POCI = posterior circulation infarcts.

^a^ Continuous variables with non-normal distribution are expressed as median (interquartile range).

P value was comparison between between patients in MODS group and non-MODS group.

Based on SOFA score, 33 (34%) patients were in MODS group and 64 (66%) in non-MODS group within 14 days after admission. (Table 1) Patients in two groups were comparable in terms of age, sex and risk factors for stroke except that there were more smokers in MODS group (51.5% vs 28.1%, *p* = 0.023). There were no significant differences in body temperature, blood pressure and blood cell count at admission. However, considering severity of stroke and systemic illness at admission, patients in MODS group had higher NIHSS score (23.48 ± 6.12 vs 19.81 ± 4.83, *p* = 0.004), higher APACHE II score (18.70 ± 5.18 vs 15.64 ± 4.36, *p* = 0.003) and lower GCS score (6.33 ± 2.48 vs 8.14 ± 2.73, *p* = 0.002). By OCSP classification, subtypes of stroke were different between two groups. Patients in MODS group presented with higher frequency of POCI (45.5% vs 25%) but lower frequency of PACI (6.1% vs 28.6%). Based on CT or MRI, patients in MODS group had higher rate of infarction in multi vascular territories (36.4% vs 10.9%, *p* = 0.003).

**Fig 1:**
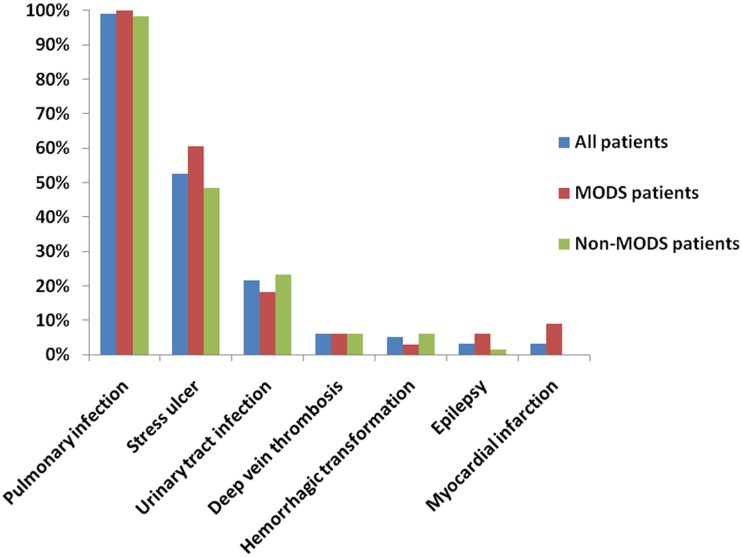
Medical complications in severe stroke patients.

Score of complications were comparable between patients in MODS group and those in non-MODS group. (Table 1) The most common complication in all patients was pulmonary infection (98.97%), which was 100% in MODS group and 98.4% in non-MODS group. (Fig 1) However, risk of myocardial infarction was lowest (3.1%), with 9.1% in MODS group and no patient in non-MODS group. The incidence of stress ulcer in all patients was 52.6%, followed by urinary tract infection (21.65%), deep vein thrombosis (6.2%), hemorrhagic transformation (5.2%) and epilepsy (3.1%), all of which had no significant differences between two groups. Patients in MODS group had higher rate of mechanical ventilation (51.5% vs 12.5%, *p* = 0.000) and osmotherapy (87.9% vs 68.8%, *p* = 0.039) in acute phase of stroke. (Table 1) Moreover, in-hospital mortality was much higher in patients with MODS (69.7% vs 9.4%, *p* = 0.000).

**Fig 2:**
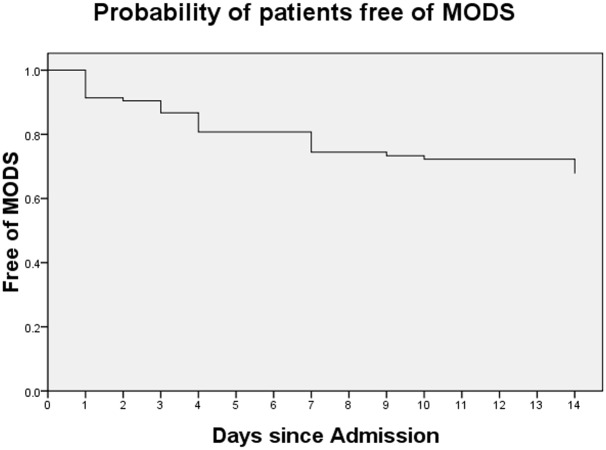
Probability of patients free of MODS.

The risk of developing MODS in severe stroke patients was higher in the first week after admission.(Fig 2) Cox regression was performed to investigate predictors for the development of MODS, with age, sex, history of smoking, subtypes of stroke by OCSP, infarction of vascular territory (single or multi), NIHSS score, GCS score and APACHE II score in the model. NIHSS score (RR = 1.084, 95% CI 1.019–1.153) and infarction in multi vascular territories (RR = 2.345 95% CI 1.105–4.978) were independent risk factors for development of MODS. (Table 2)

**Table 2:** Independent risk factors for development of MODS in severe stroke patients.

Baseline Variables	n	MODS		
		*β*	*p* value	Exp(B) 95% CI
NIHSS score (at admission)	97	0.081	0.010	1.084 (1.019, 1.153)
Infarction in multi vascular territories	97	0.852	0.026	2.345 (1.105, 4.978)

Cox regression was performed to find predictors for development of MODS, with age, sex, history of smoking, subtypes of stroke by OCSP, infarction of vascular territory (single or multi), NIHSS score, GCS score and APACHE II score in the model. Abbreviations: MODS = multiple organ dysfunction syndrome; NIHSS = NIH Stroke Scale.

## Discussion

To our knowledge, this is the first study concerning about MODS in acute severe stroke patients. There are three main findings in our study. First, 34% of sever stroke patients developed MODS in acute phase of stroke, and those patients with MODS had higher in-hospital mortality. Second, the risk of developing MODS was much higher in the first week after onset. Third, infarction in multi vascular territories and NIHSS score at admission predicted the development of MODS.

Studies about MODS in stroke patients are rare. In a study of 2213 acute stroke patients, the incidence of MODS was 3.3% in patients with diabetes and 1.8% in those without.[8] Another study of 1751 acute stroke patients in china reported that 11.7% of the included subjects had MODS.[9] Controversial results may be related to different MODS definitions and ethnic background. In our study, we only recruited severe stroke patients and found that 34% of subjects had MODS. Several factors may contribute to the higher incidence. First, all patients in our study may be more likely to meet the diagnosis of MODS as decreased level of consciousness at admission. Second, severe stroke patients may have higher risk of medical complications because of brain edema, severe disability and aspiration.[6] Third, systemic inflammatory response may be more serious in severe stroke patients which could cause the development of MODS. In addition, we also found that in-hospital mortality was much higher in patients with MODS than that in non-MODS group, which imply that early identification and treatment of MODS in severe stroke patients is critical important.

We found that the risk of developing MODS was much higher in the first week after onset, which is associated with brain edema, dysfunction of respiratory and cardiovascular system. Cerebral edema tends to peak within the first 3 or 4 days after stroke onset and leads to decreased level of consciousness and GCS score.[1, 17] It had also been reported in our previous study that the risk of fever and chest infection was the highest on the seventh day after onset.[15] Old age (>65 years), speech impairment, severity of post-stroke disability, cognitive impairment, and dysphagia had been reported as predictors for the development of pneumonia in a prospective cohort study with stroke patients.[18] For cardiac complications, results from a study with 846 stroke patients had shown that hazard of a first serious cardiac adverse events peak day 2 and 3 after acute stroke.[19] It is estimated that occurrence of cardiac adverse events may be related to stroke-induced autonomic dysregulation and physiological stress response. Therefore, these results imply that clinicians should pay more attention to severe stroke patients in the first week after onset because of high risk of medical complications and MODS.

Clinical outcome and their predictors in severe stroke patients had been wildly investigated. In a study of 243 severe stroke patients (NIHSS≥ 20), favorable outcomes (modified Rankin scale score of ≤ 3) were predicted by lower age, preceding cerebrovascular events, hypolipemic pretreatment, lower acute temperature, lower sub-acute glucose concentration and spontaneous or treatment-induced recanalization.[2] While in another study of 850 stroke subjects admitted to ICU, 3-month mortality and poor outcome were predicted by initial stroke severity and dependence on a ventilator.[20] Moreover, initial NIHSS score was also associated with 30 day mortality in studies of MI-MCA.[21, 22] In our study, we demonstrated that NIHSS score was a predictor for MODS, strengthening the result that stroke severity is a predictor for clinical outcome in severe stroke patients. We also found that infarction in multi vascular territories was an independent risk factor for MODS. This may be explained by the fact that infarction in multi vascular territories could increase the risk of fatal brain edema. It had also been reported that infarction in multi vascular territories could predict mortality in patients with severe MCA stroke.[21] However, we did not find relationship between diabetes mellitus and MODS. Future studies with large sample size are required to clarify this association. Although MODS group had more smokers, history of smoking was not an independent risk factor for development of MODS in our study. This can be explained by that smoking can cause arteriosclerosis and ischemic stroke, while the effect of smoking on MODS may be covered by infarction in multi vascular territories in Cox regression model.

Pulmonary infection and stress ulcer were the most common complications for severe stroke patients, which is consistent with previous studies.[15, 23] However, their incidence was much higher in severe stroke patients. Our results also confirm that there are relatively low frequencies of poststroke seizures and deep vein thrombosis.[23] However, we did not find the relationship between complication score and MODS, suggesting that number of complications may have little association with the development of MODS in severe stroke patients.

The strengths of our study include our prospective recruitment and homogeneity of contained patients. In previous studies, participants usually included both ischemic and hemorrhage stroke patients or all stroke patients admitted to ICU.[24, 25] We also investigated possible predictors for MODS in different aspects including clinical, biological and radiologic parameters. This result would provide important information for clinical management of severe stroke patients.

Several limitations should be considered in our study. First, this study was conducted in a single center with inevitable selective bias and did not triage criteria by hemisphere (such as DESTINY II: NIHSS>14 in patients with infarcts of the nondominant hemisphere and NIHSS>19 in patients with infarcts of the dominant hemisphere).[26] However, we recruited all patients who met our including criteria for 4.5 years to provide valuable information. Second, SOFA was not conducted everyday within acute phase of stroke. This is related with the properties of our observational study as it was not necessary to take biological tests everyday in many patients. Third, relationship between etiologies of stroke and MODS were not assessed in our study. It has been reported that severe strokes seem to be more frequently caused by cardiac emboli and less frequently caused by large artery occlusive mechanisms.[2] In addition, we only investigate risk factors for MODS within 14 days after admission. Long-term clinical outcomes were not observed in this study. Future cohort studies are required to identify the association between MODS and long-term clinical outcome. Finally, results in this study should be interpreted with cautions because of our only included severe stroke patients, this limitation may reduce the external validity of our study.

## Conclusions

In acute phase of stroke, 34% of sever stroke patients developed MODS. Infarction in multi vascular territories and NIHSS score at admission were independent risk factors for MODS. Patients with MODS had higher in-hospital mortality, suggesting that early identification of MODS is critical important.

## Supporting Information

S1 File STROBE_checklist_v4_combined_PlosMedicine. (DOCX)
